# Excision versus division of Müllerian duct remnants in male disorders of sexual development and differentiation: a prospective study to generate anatomical assessment criteria

**DOI:** 10.1007/s00383-025-06079-7

**Published:** 2025-07-30

**Authors:** Mohamed Sayed Abd El-Monsif, Ahmed Mohamed Kadry Wishahy, Noha Arafa, Mohamed Magdy Elbarbary, Gamal Eltagy, Mahmoud Marei Marei

**Affiliations:** 1 Paediatric Surgery Section/Units, Department of Paediatric Surgery, 4th Floor, Cairo University Specialised Paediatric Hospital (CUSPH) and Cairo University Children’s Hospital (Abu El-Reesh El-Mounira), Cairo University Hospitals and Faculty of Medicine (Kasr Alainy), 1 Aly Pacha Ibrahim Street, Kasr Al-Ainy Region, 11562 Cairo, Egypt; 2 Cairo University, Giza, Egypt; 3 Paediatric Surgery Department, Ghamra Military Hospital, Armed Forces, Cairo, Egypt; 4 Diabetes Endocrine and Metabolism Paediatric Unit (DEMPU), Department of Pediatrics, Abu El-Reesh El-Mounira (Cairo University Children’s Hospital), Cairo University Hospitals and Faculty of Medicine (Kasr Alainy), Al-Rashidi Street, El-Moneira District, 11562 Cairo, Egypt; 5 Paediatric Surgery Section/Units, Surgical Head Offices, 2nd Floor, Cairo University Specialised Paediatric Hospital (CUSPH) and Cairo University Children’s Hospital (Abu El-Reesh El-Mounira), Cairo University Hospitals and Faculty of Medicine (Kasr Alainy), Al-Rashidi Street, El-Mounira District, 11562 Cairo, Egypt

**Keywords:** Disorders of sexual development, Differences of sexual differentiation, Intersex, Müllerian duct remnants, Ovotestis, Gonadal dysgenesis, Persistent Müllerian duct syndrome, 46, XY DSD, External masculinisation score, Under-virilisation, Under-virilisation, Masclinising surgery, Masculinizing surgery, Gender assignment, Gender reassignment, Gender-conforming reconstructive surgery

## Abstract

**Background:**

Müllerian duct remnants (MDRs) in male disorders of sexual development (DSD) exhibit variable degrees of development and Wolffian duct relations, influencing surgical decision-making.

**Objectives:**

This study introduces the G–V_D_–M_DR_ classification to guide excision versus division decisions. It also examines the correlation between the external masculinisation score (EMS) and surgical approach for improved preoperative planning.

**Methods:**

A cohort of 18 male-reared DSD patients with MDRs was prospectively managed laparoscopically, out of 20 initially considered cases. Surgical approaches included either longitudinal splitting/division or near-total excision. G–V_D_–M_DR_ classification was introduced, which categorises MDRs based on three anatomical parameters: (A) gonadal status (G0–G3), (B) relation to the vasa deferentia (V0–V2), and (C) degree of Müllerian development (M1–M5). In addition, the predictive value of the EMS in determining surgical feasibility was evaluated.

**Results:**

Patients with higher EMS (≥ 8.5) were more likely to require MDR division due to complex MDR–vas deferens relations, while those with EMS ≤ 5.5 underwent excision. Bilateral MDR–vas deferens relations favoured division (83.3%), whereas unilateral or absent MDR–vas deferens relations were more common in the excision group (41.6% and 50%, respectively). A significant correlation was found between EMS and surgical approach (*p* = 0.004).

**Conclusion:**

The G–V_D_–M_DR_ classification, combined with EMS assessment, offers a structured and predictive framework for optimising MDR surgical management. The need to achieve single-stage, tension-free orchidopexy and the anatomical relationship between MDRs and the vas deferens govern the choice between excision and division.

**Supplementary Information:**

The online version contains supplementary material available at 10.1007/s00383-025-06079-7.

## Introduction

The management of disorders of sexual development and differentiation (DSD) in newborns, children, and adolescents presents a significant challenge in paediatric surgery, necessitating a multidisciplinary approach for optimal outcomes [1, 2]. Early and accurate diagnosis is critical in guiding gender assignment and formulating an individualised management plan that considers both anatomical and functional implications [3]. Given the complexity of these conditions, timely intervention is essential to prevent potential complications and ensure the best possible quality of life for affected individuals [4].

MDRs in male DSD pose a significant challenge due to their variable anatomical presentations and impact on surgical decision-making [3]. The presence of MDRs is attributed to abnormalities in the production or action of anti-Müllerian hormone (AMH) and/or its receptor [5], leading to incomplete regression of the paramesonephric ducts [6, 7]. This results in structures ranging from rudimentary vestiges to fully developed uteri with Fallopian tubes, which may complicate male reproductive anatomy and testicular positioning/descent [8].

The classification of MDRs based on their anatomical relationship with gonads and the Wolffian duct system is essential for guiding surgical intervention [6, 9–11]. While some cases allow for near-total excision, others necessitate a more conservative approach such as longitudinal splitting or division to preserve the male reproductive tract, particularly the vasa deferentia [6, 12]. The selection between these approaches remains a topic of debate [8], with concerns regarding fertility preservation and the potential for malignant transformation of the residual Müllerian structures [13–16]. Despite that, some authors recommend not attempting to excise them [17] for fear of injuring the vasa deferentia. It is occasionally an unexpected and perplexing intraoperative experience for the general paediatric surgeon or paediatric urologist facing this uncommon situation, without a straightforward decision-making tool [3, 12, 18].

There is a debatable overlap between prominent prostatic utricles (PU) reaching up into the abdomen and the intra-abdominal Müllerian duct remnants (MDRs) [19]. For clarity, the anomaly studied herein, in this report, is exclusively the remnant of the intra-peritoneal Müllerian ducts (i.e. above the peritoneal reflection), due to failure of regression of the Müllerian duct structures, which are normally destined to become the upper vagina, cervix, uterus and Fallopian tube. On the other hand, a PU is morphologically an outpouching/diverticulum from the posterior urethra and has been frequently managed by laparoscopic excision under cystoscopy guidance [20–22], provided it is significant enough to warrant this, according to the Ikoma classification [9, 23]

To address this gap, this study aimed to reach and introduce a novel classification system, henceforth designated the G–V_D_–M_DR_, to guide decision-making between excision and division, as an objective framework, incorporating three key anatomical parameters to evaluate the MDR development and the relationship between MDRs and adjacent structures, (1) gonadal status, (2) relation to vasa deferentia, and (3) degree of Müllerian development, for determining the feasibility of excision versus division in MDRs. In addition, we evaluate the external masculinisation score (EMS) as an external predictor of anatomy. This system aims to facilitate intraoperative/laparoscopic planning and surgical decision-making to preserve reproductive potential (whenever possible).

## Patients and methods

### Study design and population

This non-randomised prospective clinical trial was conducted over a two-year period, with subsequent cross-sectional analysis. The study initially included 20 DSD patients with MDRs, either 46, XY DSD, chromosomal DSD, or testicular/ovotesticular variants of 46, XX DSD, recruited from the specialised multidisciplinary DSD service of Cairo University Children’s Hospitals cluster, who underwent laparoscopic surgical intervention. Eighteen cases were managed with either longitudinal splitting/division or near-total excision. Two cases were subsequently excluded from surgical management for the MDR, for the reasons displayed in the “Results” (vide infra).

### Ethical approval and consent

The study was approved by the Research Scientific and Ethical Committees of Cairo University, Faculty of Medicine (the hosting institution), document reference no. CMDRF-132701. Informed written consent was voluntarily obtained from parents, ensuring a clear explanation of the planned procedures, associated risks, and permission to use collected clinical data for research. In addition, children above 7 years provided assent through a simplified form (information leaflet) that described the surgical procedure in an age-appropriate manner, considering their cognitive and emotional well-being.

### Inclusion and exclusion criteria, case recruitment and data collection

Inclusion criteria comprised cases from birth to 16 years with suspected persistent Müllerian duct remnants  or gonadal abnormalities, eligible for diagnostic or therapeutic laparoscopy. Patients were excluded if they were unfit for surgery due to poor general health or had congenital adrenal hyperplasia.

The recruited cases were encountered from cases referred for a laparoscopy, through a joint DSD clinic and interdisciplinary service. The cases were enrolled with the prospective intent of utilisation of their data for achieving the primary and secondary outcome parameters.

An initial diagnostic laparoscopy was performed for all cases, which occasionally included a gonadal biopsy. Subsequently, the hormonal profile, karyotyping, laparoscopic information (± photography), and histopathological information (whenever present) were analysed in a multidisciplinary setting, and further management was decided. Each patient underwent two separate surgical procedures, and usually 2–4 months elapsed between both procedures to permit the multidisciplinary decision process including the histopathological results, as well as counselling the family through a clinic review, on which the second procedure and the choice of excision vs. division was dependent. Based on the anatomical insights gained herein, we believe that a single surgical session can now be planned for most patients, unless a gonadal biopsy is required to inform gonadal management, see “Intraoperative determinants of surgical decision”. Data from all sources were documented prospectively and then analysed retrospectively.

### Primary and secondary outcome parameters

Primary outcome—describing the anatomical nature of MDRs and their relation to the internal genital anatomy, i.e. the male duct system (vas deferens), and the gonads, as well as the external genital anatomy, represented by the EMS assessment.

Secondary outcome—to propose optimised selection criteria to determine the most appropriate laparoscopic procedure (excision vs. division) based on anatomical and pathological findings.

### Preoperative assessments

A comprehensive preoperative assessment was conducted, including a detailed history covering age, principal complaint at presentation, initial sex assignment, consanguinity, family history of similar conditions, and any previous surgical procedures. A thorough genital examination was performed, assessing gonadal position (palpation), the number and position of orifices, phallic/clitoral size and length, and labioscrotal fold differentiation. Based on these findings, the EMS was calculated to evaluate the degree of virilisation.

The EMS is a validated tool used to objectively quantify the degree of masculinisation of the external genitalia in newborns with DSD. It provides a composite score, out of 12, based on four anatomical domains: phallic size (scores 0–3), position of each gonad (right and left, each scores 0–2), degree of labioscrotal fusion (scores 0–2), and location of the urethral meatus (scores 0–3). Each feature is assessed clinically, and the individual scores are summed to produce a total score. A higher EMS reflects greater masculinisation, with 12 representing the typical male phenotype. Most healthy-term male neonates score 12, while progressively lower scores indicate varying degrees of hypo/under-virilisation and help identify cases requiring further evaluation or intervention [2, 24]

In addition, karyotyping and hormonal profile data, including the anti-Müllerian hormone (AMH) levels, were retrieved from medical records; see Supplemental Material—ST2. Radiological findings, such as ultrasonography or MRI, were also documented to complement the diagnostic evaluation.

### Surgical decision process and intraoperative management

The approach as to whether to perform an excision or division of the Müllerian duct remnant, for each patient, was based on the following criteria, in order of priority, (1) achieving full gonadal descent into the scrotum, whenever a gonad is to be preserved; (2) protection/sparing of the vas deferens involved and fertility preservation whenever feasible; (3) excision of the Müllerian remnant whenever safe to achieve, or division to satisfy the previous two points (1) and (2), if excision is not feasible; and finally (4) debulking of the Müllerian remnant, at least centrally [25], and removal/cauterisation of its lumen/lining during the division, when opted for.

Surgical management involved a laparoscopic approach with standardised 5-mm port placement (umbilicus, right iliac fossa and left iliac fossa), and visualisation using a 30-degree angled scope and insufflation pressure of 10–12 mmHg. The laparoscopic setup is shown in Supplemental Material—SF1. Biopsy was performed for abnormal gonads, and orchidopexy was conducted in cases with normal gonads (testis/testes). Gonadectomy was reserved for dysgenetic, ovotesticular, or gonads deemed precancerous or non-functional.

Near-total excision was done as distal as possible just above the point of vas deferens entry to spare it; however, in cases with bilateral absent relation with the vas deferens total excision can be done. It was done in cases with a clear course of the vas deferens that facilitates prevention of injury, especially cases with unilateral relation between the MDR and the vas deferens or bilateral relation with the vas deferens, while one of them connected to undesired gonad (i.e. dysgenetic) for gonadectomy, as in MGD, or bilateral absent relation with the vas deferens. The MDR was mobilised from all surroundings, including the supporting ligaments of the uterus, when present, i.e. broad ligament leading to the cardinal ligaments, the round ligament and ovarian ligaments, and the peritoneal reflections; see Supplemental Material—SV for an operative video demonstration. If the lower end was communicating with the urethra, an endoloop ligature is placed, akin to Lima et al. [26, 27].

Division or longitudinal splitting was done in the midline of the structure, down to the peritoneal reflection, using an energy device as a diathermy hook, harmonic scalpel or LigaSure™ in cases with unclear course of the vas deferens or complex relation between the MDR and the vas deferens, especially if bilateral, as in PMDS or high point of vas deferens entry. It was also done in cases associated with an underdeveloped elongated MDR having the vas deferens running over a long length of the wall, especially if bilateral; see Supplemental Material—SV (operative video).

### Generation of the anatomical classification system (G–V_D_–M_DR_) and statistical analysis

Subsequent to performing either an MDR excision or division, based on the four criteria displayed above, cases were reviewed in retrospect to find associations between the decision dictated by this order of prioritisation and the anatomical findings.

Data analysis was performed using SPSS software (version 27.0, SPSS Inc., Chicago, Illinois, USA). Quantitative variables were described as mean ± SD, median, minimum and maximum, compared using the independent *T*-test for any 2 independent groups, with a significant *p* value at *p* < 0.05. Qualitative variables were described as frequency and percentage. Comparison for qualitative variables was done using the Chi-square test and Fisher’s exact test.

## Results

### Clinical presentations

A description of the studied cohort and a layout of the various clinical presentations is displayed in Table 1. Seventeen cases (85%) presented with hypospadias, which was mid-penile or more proximal. All 20 cases (100%) presented with undescended testes, either unilateral or bilateral, and palpable or impalpable. One case (5%) presented with bilateral palpable testicles without hypospadias. Two cases (10%) presented with a unilateral palpable undescended testis and a penoscrotal hypospadias. No cases presented with an isolated unilateral undescended testis or an isolated hypospadias. The details of the clinical examination data are appended in Supplemental Material—Table ST1.

**Table 1:** Patients’ demographics and characteristics

Patients’ demographics and characteristics		Total (*N* = 20)
Age (months) at first intervention	Median (IQR)	17 (8–24)
	Range	3–108
Clinical presentations^‡^ (initial)	Ambiguous genitalia	6 (30%)
	Bilateral UDT	5 (25%)
	Unilateral UDT	5 (25%)
	Recurrent UDT after inguinal approach for an UDT (initially impalpable)	1 (5.0%)
	Accidental discovery of MDR during inguinal surgery for (a) palpable gonad(s)	3 (15.0%)
Karyotyping^‡^	46, XY	14 (70.0%)
	46, XX	2 (10.0%)
	46, XY/46, XX (chimeric)	1 (5.0%)
	46, XY/45, XO	3 (15.0%)
Consanguinity	Positive	8 (40%)
	Negative	12 (60%)
Similar conditions within the family	Positive	2 (10.0%)
	Negative	18 (90.0%)
Drug administration to the mother during pregnancy	Gestagens	8 (40.0%)
Associated medical co-morbidities	Cardiac anomalies	1 (5.0%)
	G6PD	1 (5.0%)
	Renal anomalies	2 (10.0%)
	None	14 (80.0%)
Maternal history of recurrent abortion	Positive	7 (35.0%)^*^
	Negative	13 (65.0%)

Data presented as number (percentage) or median (IQR) and range. Percentages are calculated in reference to all initially enrolled cases (N = 20)

*IQR* interquartile range, *UDT* undescended testis/testes, *G6PD* glucose 6-Phosphate dehydrogenase deficiency

^*^Four cases (20%) had a 46, XY karyotype, two cases (10%) had a 46, XY/45, XO karyotype and one case (5%) was chimeric

^‡^See (Supplemental Material—Tables ST1 and ST2)

### External anatomy

The mean EMS was significantly lower in the excision group (5.33 ± 1.98), compared to the division group (8.58 ± 1.74). This difference was statistically significant (*t* = − 3.403, *p* = 0.004).

The Prader score had a mean ± SD of 3.50 ± 1.00, range: 2–5. The Quigley scale had a mean ± SD of 2.95 ± 1.08, range: 1–4.

Our findings suggest a correlation between the EMS and the choice between excision and division of MDR. Cases with EMS ≥ 8.5 were found suitable for division, whereas those with EMS ≤ 5.5 were found more suitable for excision, as displayed in Table 2.

**Table 2:** Excision vs division regarding the external genital configuration

External genital examination		Excision (*n* = 12)	Division (*n* = 6)	Test value	*P* value	Sig.^†^
Hypospadias	Present	12 (100.0%)	3 (50.0%)	7.200^*^	0.007	HS
	Absent	0 (0.0%)	3 (50.0%)			
Labioscrotal folds	Fused	5 (41.7%)	6 (100.0%)	5.727^*^	0.017	S
	Bifid scrotum	7 (58.3%)	0 (0.0%)			
EMS	Mean ± SD	5.33 ± 1.98	8.58 ± 1.74	− 3.403^#^	0.004	HS
	Range	2–8.5	7–10.5			

Data presented as number (percentage) or Median ± SD and range. Percentages are calculated in reference to the subgroup, i.e. Excision = 12 patients and Division = 6 patients

*EMS* external masculinisation score, *SD* standard deviation

^†^Sig.: *p*-value > 0.05: Non-significant (NS); *p*-value < 0.05: Significant (S); *p*-value < 0.01: Highly significant (HS); ^*^Chi-square test; ^#^Independent *t*-test

### Internal anatomy

The relationship between MDRs and the vas deferens differed between the excision and division groups. Cases with bilateral undescended gonads and bilaterally present MDR–vas deferens relations were more frequent in the division group (83.3%), compared to the excision group (8.3%). Other MDR–vas deferens relation patterns were predominantly observed in the excision group, particularly cases with bilaterally absent MDR–vas deferens relations (50%) and unilaterally present MDR–vas deferens relations (46.6%), as displayed in Table 3.

**Table 3:** Excision vs. division regarding the internal genital configuration: relationship between the MDR and vas deferens

Relationship between the MDRs and the male duct system	Excision (*n* = 12)	Division ( = 6)
Unilateral undescended gonad with unilaterally present relation between its vas deferens and the MDR, and absent relation of the MDR with the vas deferens of the scrotal gonad	1 (8.3%)^#^	None
Unilateral undescended gonad with unilaterally present relation between the MDR and the vas deferens of the scrotal gonad only	2 (16.6%)^#^	None
Unilateral undescended gonad with bilaterally absent relation between the MDR and the vas deferens of either the undescended or the scrotal gonads	3 (25.0%)^❖^	None
Unilateral undescended gonad with bilaterally present relation between the MDR and the vas deferens of both the undescended and the scrotal gonads	None	1 (16.66%)
Bilateral undescended gonads with bilaterally present relation between the MDR and the vasa deferentia of both undescended gonads	1 (8.3%)^*^	5 (83.3%)^*^
Bilateral undescended gonads with unilaterally present relation between the MDR and the vas deferens of only one of the undescended gonads	2 (16.6%)^#^	None
Bilateral undescended gonads with bilaterally absent relation between the MDR and the vas deferens of either of the undescended gonads	3 (25%)^❖^	None

Data presented as number (percentage)

*MDR* Müllerian duct remnants

^*^Chi-square test, *p*-value = 0.027 (significant). Test value = 14.25

^#^Unilaterally present relation between the MDR and the vas deferens (of one gonad)

^❖^Bilaterally absent relation between the MDR and the vasa deferentia; Percentages are calculated in reference to the subgroup, i.e. Excision = 12 patients and Division = 6 patients

### Gonadal considerations

Division increased the lateral mobility of the mesorchium of the intra-abdominal gonads; see Supplemental Material—Table ST3, resulting in a statistically significant difference between the excision and division groups, regarding the possibility of performing a unilateral single-stage orchidopexy, being more with division, i.e. 5 cases (83.33%) of division (out-of-6) versus 4 cases (33.33%) of excision (out-of-12), (*p* value = 0.046). There was a statistically significant difference between cases which needed excision and cases that needed division, regarding the relation between the MDR and the vas deferens, with cases having bilateral undescended testis and a bilateral relation between the MDR and the vas deferens more with division (*p* value = 0.027). We performed six laparoscopic gonadectomies, two of them were from the right side and the other four were from the left side. Gonadal management is displayed in Table 4.

**Table 4:** Laparoscopic gonadectomy vs. orchidopexy

Laparoscopic procedures for intra-abdominal gonads^‡^		Number of gonads
Gonadectomy	Gonadectomy by inspection without biopsy (one dysgenetic ovary + one dysgenetic ovotestis)	2 (5.55%)
	Gonadectomy for dysgenetic testis	1 (2.77%)
	Gonadectomy for ovary	1 (2.77%)
	Gonadectomy for dysgenetic ovary	1 (2.77%)
	Gonadectomy for ovotestis	1 (2.77%)
Orchidopexy	Single-stage (primary) orchidopexy	9 (25%)
	FSO (Fowler–Stephens orchidopexy)	4 (11.11%)
	Staged traction	2 (5.55%)

Data presented as number (percentage). Percentages are calculated in reference to all surgically managed MDRs, i.e. excision or division (N = 18)

^‡^Remaining gonads were absent or inguinoscrotal, see Supplemental Material—Table ST3

Required inguinal or scrotal procedures for palpable gonads were two gonadal biopsies (5.55%) from the right side; two gonadectomies (5.55%) for right and left ovaries and their tubes; and one orchidopexy (2.77%) through scrotal incision for right recurrent undescended testis.

### ***The proposed anatomical classification system (G–V***_***D***_***–M***_***DR***_***)***

A novel classification system, G–V_D_–M_DR_, was developed to categorise MDRs based on three key parameters: (1st) gonadal status: G0 (absent gonad), G1 (dysgenetic/ovary), G2 (ovotestis), G3 (normal right/left testis); (2nd) relation to vasa deferentia: V0 (absent relation bilaterally), V1 (unilateral relation), V2 (bilateral relation); and (3rd) degree of Müllerian development: M1–M5 ranging from rudimentary MDRs (M1) to a uterus with bilateral Fallopian tubes (M5). The correlation between EMS and surgical approach was also analysed.

**Fig. 1:**
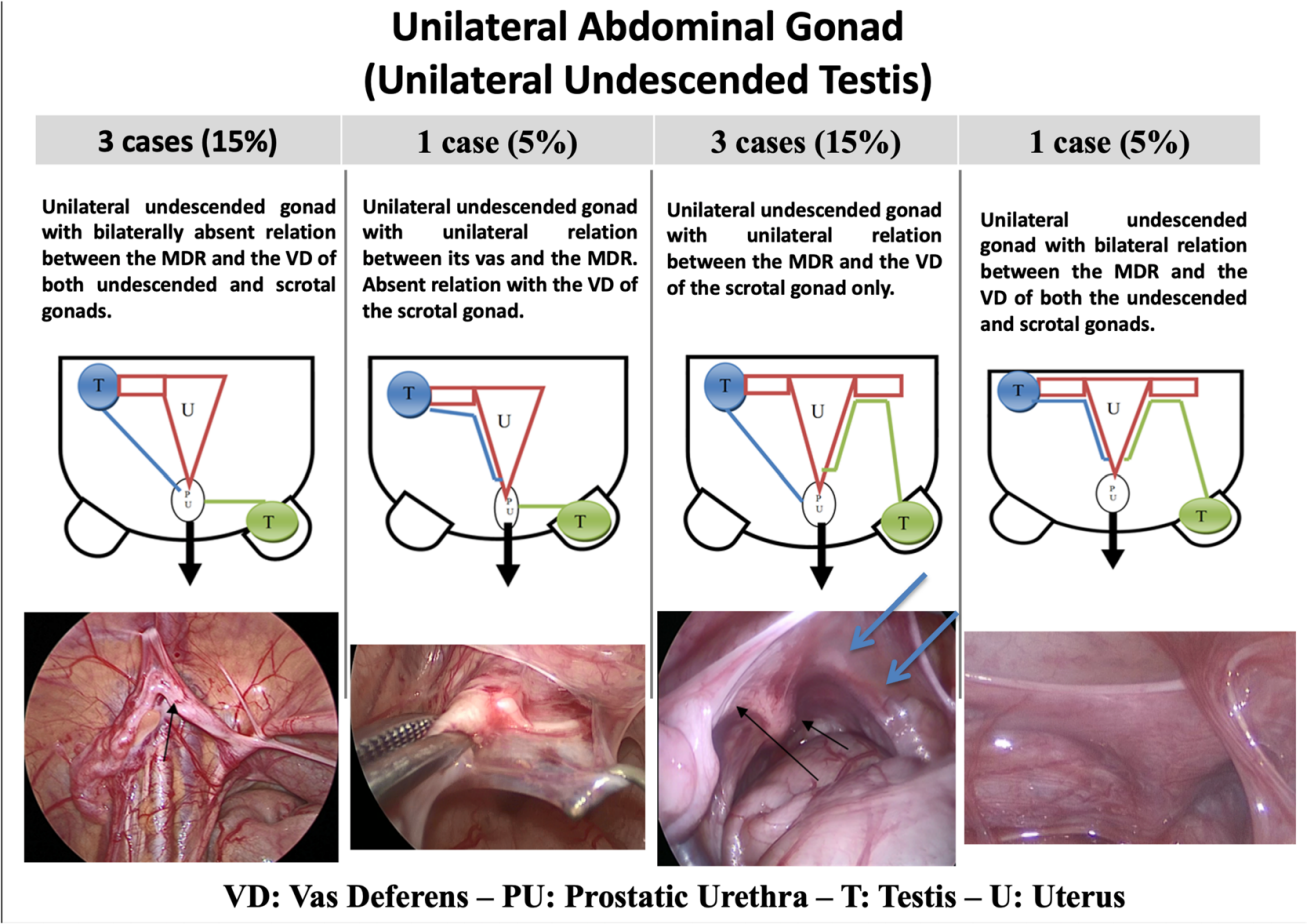
Configurations associated with unilateral undescended gonads (*diagrammatic and operative demonstration*)

**Fig. 2:**
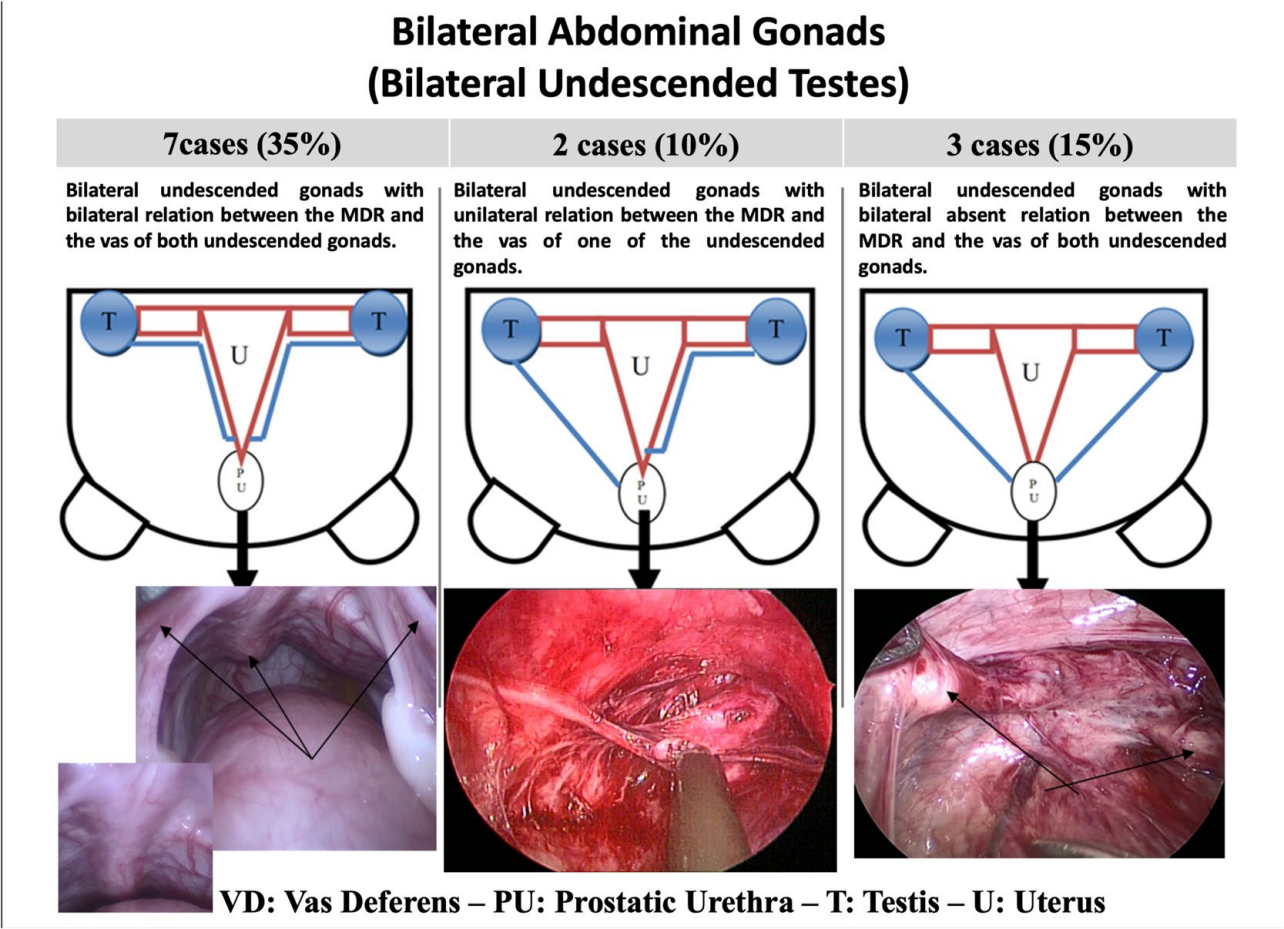
Configurations associated with bilateral undescended gonads (*diagrammatic and operative demonstration*)

The proposed classification reflects MDR management based on gonadal status, vas deferens relation, MDR characteristics, karyotype, and vas deferens entry point. Near-total excision is preferred in cases with absent/undesired gonads (G0/G1), absent/unilateral vas deferens relation (V0/V1), and limited MDR structures' development (M1—M3), while division or longitudinal splitting is more suitable for normal/desired gonads (G3), bilateral vas deferens relation (V2), and extensive MDR development and involvement (M4—M5). In addition, a higher vas deferens entry point into the MDR favours division over excision (Table 5; Figs. 1 and 2).

**Table 5:** Proposed selection criteria based on the novel classification

	Near-total excision	Division or longitudinal splitting
Gonads	Absent gonad (G0) Undesired gonad (G1)—due to decreased demand to preserve the ipsilateral vas deferens	Normal or desired testicular tissue (G3)—due to increased demand to preserve the vas deferens
Vas deferens relation	Absent relation with the vas deferens (V0) Unilateral relation with the vas deferens (V1)	Bilateral relation with the vas deferens (V2)
MDRs	Fallopian tube alone, e.g. ovotesticular DSD: (M1) Uterus alone, e.g. MGD: (M2) Uterus and unilateral fallopian tube e.g. MGD or ovotesticular DSD: (M3)	Bilateral Fallopian tubes, e.g. PMDS (M4) Uterus and bilateral tubes, e.g. PMDS (M5)
Karyotyping	46, XY/45, XO MGD 46, XY MGD Ovotesticular DSD (raised as male)	PMDS (46, XY) XX testicular DSD or XX male (raised as male)
Point of vas entry	A low point of vas deferens entry into MDR	A high point of vas deferens entry into MDR

*MDRs* Müllerian duct remnants, *DSD* disorders of sexual development, *MGD* mixed gonadal dysgenesis, *PMDS* persistent Müllerian duct syndrome

MDRs were managed through near-total excision when the vas deferens had a clear course (V1) or when one side was associated with an undesired gonad (G1). In cases with complex MDR–vas deferens relations (V2), longitudinal splitting/division was preferred to preserve reproductive structures. Table 6 summarises the comparison between both techniques and their outcomes.

**Table 6:** Summary comparison of clinical and intraoperative parameters between the excision and division groups

Parameter	^*^Excision (*n* = 12)	^*^Division (*n* = 6)	*p* value	Remarks
Mean EMS (± SD)	5.33 ± 1.98	8.58 ± 1.74	0.004	Higher EMS favours division
Bilateral MDR–vas deferens relation	1 (8.3%)	5 (83.3%)	0.027	Strongly favours division
Single-stage orchidopexy	4 (33.3%)	5 (83.3%)	0.046	Division improves mesorchial mobility
Gonadectomy performed	4 (33.3%)	2 (33.3%)	NS	Not significantly different
UTI/epididymitis history	4 (33.3%)	2 (33.3%)	NS	Similar postoperative early complications
Intraoperative complications	0	0	–	None in either group
Indications for choice	Low EMS, unilateral/absent vas relation, undesired gonad	High EMS, bilateral vas relation, desired gonads	–	Based on G–V_D_–M_DR_ criteria

NS: Non-significant

^*^Division was associated with higher EMS, more frequent bilateral vas deferens involvement, and improved feasibility of single-stage orchidopexy

### Cases excluded from surgical excision/division

No surgical management was done in two patients. One case: the utricle was extremely small and could not be visualised without incising the retro-vesical region, and the vas deferens opened into its apex. One case: 46, XX ovotesticular DSD with bilateral ovotesticular tissue, bilateral vas deferens and a uterus with bilateral tubes. This patient had low basal androgens, EMS of 1, Prader’s score of 2, and the decision was made to be raised as a female.

### Gender assignment/reassignment

This study initially included 20 cases, of which 15 (75%) were initially raised as males with no subsequent change. Four cases were initially reared as females, and after verification of all genotypic and phenotypic data were subsequently reared as females. One case went the opposite way, which was explained above.

### Early surgical outcomes and follow-up

No intraoperative complications, neither general complications (bleeding, hypercapnia or conversion to open), nor specific (ureteric or intestinal injury) were encountered or found. Two-week follow-up: no port site infection, hematoma or hernia were encountered. Three- and six-month follow-ups: No port site hernia, recurrent undescended testis, UTI or epididymo-orchitis were encountered.

Six months after laparoscopy, we performed a staged hypospadias repair (employing preputial grafts) for four patients from the studied group. Following the second-stage repair, only one patient (25%) presented with symptoms suggestive of an upper UTI; however, this patient had left hydronephrosis due to pelviureteric junction obstruction on the same side of the undetectable vas deferens, prior to any hypospadias repair, so the UTI may be multifactorial.

## Discussion

This is one of the largest series addressing the surgical management of Müllerian duct remnants, to date. This is an area where literature is sparse, and neither comparisons between the two techniques nor selection criteria for them have been published [8], and most reports (especially about division) are singular cases [28]. In this study, we introduced a novel classification system, the G–V_D_–M_DR_, to guide the surgical management of MDRs in male patients with DSD. Setting the scene and context for this work, it is important to note that, intuitively, an excision is ideal for those cases, being raised as males, thus not needing this structure, and owing to the potential concerns around the MDR presence, i.e. including the potential for malignant degeneration [7, 29, 30], voiding problems [26], urinary tract infection and epididymitis/epididymo-orchitis [6, 27] (which was present in 6 of our cases) and anchoring undescended testes abdominally [9, 25, 30, 31] (which was unanimously present in all our cases); however, the trade-off of accepting a division may be dictated by the configuration [3, 12, 28, 32]. Our results indicate that bilateral MDR–vas deferens relations and higher EMS favour division, whereas cases with absent/unilateral MDR–vas deferens relations and lower EMS scores are more suited for near-total excision. The overall comparative outcomes between both groups are summarised in Table 6, illustrating the anatomical and functional rationale guiding each surgical approach. A survey of the available literature revealed that no unified anatomical scoring system currently exists to guide the surgical management of MDRs. Instead, reported approaches are often based on individual surgeon preference or intraoperative judgement, reflecting underlying anatomical principles but lacking standardisation. In this context, our proposed classification system seeks to consolidate these anatomical considerations into a structured and reproducible framework to support more consistent, evidence-based decision-making.

### Comparison of both performed techniques in the current literature

In our study, 12 cases (67%) underwent MDR excision, aligning with the approach of Wei et al, done successfully in three cases [33]. Farikullah et al also recommended excision to achieve an orchidopexy while minimising the risk of malignancy [30] which they have done successfully in eight cases. On the other hand, some surgeons/authors, for the concern of damaging the vas deferens during its mobilisation from the MDR opt for leaving them in situ, as suggested by Vandersteen et al [17]; this argues the case for division or longitudinal splitting, consistent with the approach reported by El-Gohary, on 5 cases, where longitudinal splitting was successful to enable orchidopexy reaching the scrotum [34]. We have adopted this in 6 cases (33%). Raicevic et al [8] did an extensive systematic literature review to determine surgical outcomes, to find 10 articles over 10 years, reporting on 23 patients, where complete removal was done in 9 cases, near-complete removal in 11 cases and longitudinal splitting in 1 case, all reporting good outcomes. In recent years, robot-assisted techniques have also been employed for MDR excision in paediatric patients, offering improved dexterity and 3D visualisation, which may be particularly beneficial in cases with complex vas deferens relations [27, 35]. While not utilised in our cohort, robotic surgery may represent a valuable option in selected cases and has been increasingly adopted in other paediatric urological procedures [27, 35]. If a PU is involved, laparoscopic excision under cystoscopy guidance [20–22] could be considered.

### Preoperative predictors of surgical decision

#### Utilising the EMS preoperatively

The mean EMS was significantly higher in cases that required MDR division (8.58 ± 1.74) compared to those that underwent excision (5.33 ± 1.98). Our findings suggest that a higher EMS correlates with (a) normal/adequate development of the male duct system (vas deferens), (b) a more complex MDR–vas deferens relationship, and c) a higher incidence of bilaterally normal gonads, making excision less safe. Therefore, a higher EMS favoured MDR division as the safer and more feasible option. In cases with 5.5 < EMS < 8.5, the decision cannot be reliably predicted before diagnostic laparoscopy. This observation can be attributed to testosterone production by Leydig cells, which is not only responsible for external genital virilisation but also plays a crucial role in mesonephric duct differentiation into the vas deferens [36].

Based on this correlation/relationship, we propose a surgical decision-making algorithm using EMS as a preoperative predictive tool. If the EMS is greater than or equal to 8.5, the case is usually suitable for division. If the EMS falls between 5.5 and 8.5, the surgical approach cannot be reliably determined preoperatively and awaits the diagnostic laparoscopy. If the EMS is less than or equal to 5.5, the case is usually suitable for excision if the patient is raised as a male. This would facilitate the surgeon’s preparedness preoperatively. This novel utilisation of the EMS system when retrospectively applied to the cases reported by Brandli et al [37], accurately predicted their surgical decisions. In their study, MDR division was performed in two cases of PMDS with an EMS of 10 to prevent injury or devascularisation of the male duct system, despite using a Pfannenstiel incision that could have facilitated excision, as their preference for division was guided by concerns for vas deferens preservation. Still one of their two cases needed a testicular microvascular auto-transplantation [37]. Contrarily, Wei et al [33] reported three cases of PMDS with EMS scores exceeding 10, yet still opted for excision, but had to adopt an open surgical approach, rather than laparoscopy, to achieve this.

#### Impact of karyotyping

Karyotypic subtypes associated with excision included 46, XY ovotesticular DSD, 46, XY MGD, 46, XY/45, XO MGD, and non-CAH 46, XY under-virilised males. In these groups, excision was feasible either due to absent or unilateral MDR–vas deferens relations with clearly defined courses that allowed safe dissection. These findings align with Krstić et al [38], who performed MDR excision in 3-out-of-4 cases of ovotesticular DSD assigned to the male sex, and with Tambo et al [39], who excised MDRs in 2-out-of-3 cases with 46, XY/46, XO mosaicism. Cases requiring division included 46, XY PMDS, where two patients had bilateral complex MDR–vas deferens relations, and a third had high-entry vas deferens on the left side. This finding aligns with Brandli et al [37] but contradicts Wei et al [33]. Division was also required in two non-CAH 46, XY under-virilised males with bilateral anatomically unclear vas deferens relations. In addition, in one case of 46, XX male, the MDR was elongated and closely applied to the vas deferens, precluding safe excision. See Supplemental Material—Table ST4 for further clarification, expanded and aggregate details.

### Intra-operative determinants of surgical decision

Our findings underscore that the intraoperative choice between excision and division is determined by MDR morphology, its relationship with the vas deferens, gonadal histopathology (whenever available), and prior knowledge of karyotyping and the EMS. To standardise surgical decision-making, we introduce the G–V_D_–M_DR_ classification system, a novel anatomical classification framework based on diagnostic laparoscopy and gonadal biopsy. This system categorises MDR cases using three key parameters: Müllerian development (M1–M5), ranging from rudimentary remnants to a uterus with bilateral Fallopian tubes; vas deferens relation (V0–V2), reflecting the presence and complexity of MDR–vas deferens relations; and gonadal status (G0–G3), indicating absent, normal, dysgenetic, or ovotesticular gonads. For example, a case of PMDS with a uterus and bilateral Fallopian tubes (M2), a bilateral MDR–vas deferens relation (V2), and bilaterally normal testicular tissue (G1) can be classified as M2\V2\GR1\GL1. The proposed selection criteria for the surgical approach suggest that near-total excision is preferable when the vas deferens can be dissected from the lateral wall of the MDR, following it downwards and distally. The point of vas deferens entry should be well visualised, and excision should be performed above this point to preserve the vas deferens. Division or longitudinal splitting is performed using hook diathermy or preferably the harmonic scalpel or LigaSure^™^ (or equivalent) device. With the criteria generated from this work, in most future cases, the decision between excision and division can be made preoperatively or intraoperatively without staging, except where gonadal histology is essential to guide management.

### Comparison of both performed techniques in our practice

A comparative analysis of excision and division highlights several advantages and disadvantages. Excision eliminates the risk of MDR-associated malignancy and reduces the incidence of recurrent urinary tract infections and epididymitis/epididymo-orchitis by removing lumen-bearing MDRs. However, it poses a higher risk of vas deferens injury due to increased manipulation and may fail still to achieve a complete removal, which can anchor gonads and hinder tension-free orchidopexy. Division, on the other hand, minimises vas deferens manipulation, reducing injury risk, and should decrease the risk of urinary tract infections by occluding the MDR lumen through cauterisation. In addition, it facilitates single-stage, tension-free orchidopexy by improving lateral mesorchium mobility. Nevertheless, the division procedure does not eliminate the risk of malignancy as MDR tissue is retained, necessitating vigilant follow-up with ultrasound or MRI. Electrocauterisation-induced fibrosis of the lining and lumen may hypothetically reduce malignancy risk but does not eliminate it. A detailed comparison of these outcomes is summarised in Table 6.

### Limitations of the study

Despite the promising findings, a key limitation of this study is the relatively small sample size, which may impact the generalisability of the findings, although this would be one of the largest paediatric series published in this discipline [8]. The study lacks long-term follow-up data to assess potential complications such as infertility, malignancy, and recurrence of MDR-related pathology. From an operative outcome perspective, this study reports only on the early phase of that, and an extended follow-up is still required in subsequent future work. This would include an extended follow-up for fertility potential, testicular position and occurrence of epididymitis/epididymo-orchitis. Longitudinal studies assessing post-surgical outcomes, including reproductive function and malignancy risk, are essential to establish the long-term safety and efficacy of excision versus division. In addition, the retrospective application of the G–V_D_–M_DR_ and EMS-based classification to previously operated upon cases, despite being prospectively sought for and planned, invites caution with its upcoming applicability in broader clinical settings, without a prospective trial for further validation. Future research should focus on multicentred studies with larger cohorts to validate the proposed classification system and refine its predictive accuracy.

## Conclusion

Combining the EMS with the G–V_D_–M_DR_ classification provides a comprehensive framework for determining the feasibility of MDR excision versus division. Division is favoured by a higher EMS, a more complex MDR–vas deferens relationship, and bilaterality. Excision is favoured by lower EMS scores, and unilateral MDR–vas deferens involvement. Both laparoscopic excision and division were technically feasible and resulted in favourable short-term outcomes.

## Supplementary Information

Below is the link to the electronic supplementary material.

Supplementary file 1 (DOCX 237 KB): Supplemental Figure (SF1): Laparoscopic Setup: Position of the patient and the surgical team. S: Surgeon, SA/C: Surgical assistant/Camera holder, SN:Scrub nurse, AN: Anaesthesiologist

Supplementary file 2 (DOCX 21 KB): Supplemental Table (ST1): Examination Data in All Initially Enrolled Cases.Examination Data. Data presented as number (percentage). Percentages are calculated inreference to all initially enrolled cases

Supplementary file 3 (DOCX 21 KB):  Supplemental Table (ST2): Chromosomal and Endocrinal Status of Surgically Operated Cases (N =18). Data presented as number (percentage). ^†^Sig.: *p*-value > 0.05: Non-significant (NS); *p*-value < 0.05: Significant (S); *p*-value < 0.01: Highly significant (HS); ^*^Chi-square test. Percentages are calculated in reference to the subgroup, i.e. Excision = 12 patients and Division = 6 patients

Supplementary file 4 (DOCX 21 KB): Supplemental Table (ST3): Gonadal Status and Positions by Laparoscopy. Data presented as number (percentage). Percentages are calculated in reference to all initially enrolled cases

Supplementary file 5 (DOCX 33 KB): Supplemental Table (ST4): Karyotype-Specific Surgical Decision Factors

Supplementary file 6 (MP4 88235 KB): Supplemental Video (SV): MDRs Division vs. Excision

## Data Availability

Data is provided within the manuscript and supplementary information files. The entire/unprocessed data and material for this study are available and stored confidentially and can be presented and shared upon a reasonable request to the corresponding author.

## Abbreviations

DSD: Disorders of sexual development and differentiation
G: Gonads
VD: Vas deference
MDR: Müllerian duct remnant
PU: Prostatic utricle
EMS: External masculinisation score
AMH: Anti-Müllerian hormone
PMDS: Persistent Müllerian duct syndrome
CAH: Congenital adrenal hyperplasia
MGD: Mixed gonadal dysgenesis
UTI: Urinary tract infection
UDT: Undescended testis/testes
FSO: Fowler-stephens orchidopexy
G6PD: Glucose-6-phosphate dehydrogenase
46, XX: Typical female karyotype
46, XY: Typical male karyotype
45, XO: Monosomy X
US: Ultrasonography
MRI: Magnetic resonance imaging
SPSS: Statistical package for the social sciences
SD: Standard deviation
IQR: Interquartile range
SV: Supplemental video
ST: Supplemental table
SF: Supplemental figure

## References

[1] Wolffenbuttel KP, Hersmus R, Stoop H et al (2016) Gonadal dysgenesis in disorders of sex development: diagnosis and surgical management. J Pediatr Urol 12:411–416. 10.1016/j.jpurol.2016.08.015 27769830 10.1016/j.jpurol.2016.08.015

[2] Ahmed SF, Achermann JC, Arlt W et al (2011) UK guidance on the initial evaluation of an infant or an adolescent with a suspected disorder of sex development. Clin Endocrinol (Oxf) 75:12–26. 10.1111/J.1365-2265.2011.04076.X 21521344 10.1111/j.1365-2265.2011.04076.x PMC3132446

[3] Steven M, O’Toole S, Lam JPHH et al (2012) Laparoscopy versus ultrasonography for the evaluation of mullerian structures in children with complex disorders of sex development. Pediatr Surg Int 28:1161–1164. 10.1007/s00383-012-3178-3 23064803 10.1007/s00383-012-3178-3

[4] Kim KS, Kim J (2012) Disorders of sex development. Korean J Urol 53:1–8. https://doi.org/10.4111/kju.2012.53.1.1 22323966 10.4111/kju.2012.53.1.1 PMC3272549

[5] di Clemente N, Belville C (2006) Anti-Müllerian hormone receptor defect. Best Pract Res Clin Endocrinol Metab 20:599–610. 10.1016/j.beem.2006.09.004 17161334 10.1016/j.beem.2006.09.004

[6] Krstić ZD, Smoljanić Ž, Mićović Ž et al (2001) Surgical treatment of the Müllerian duct remnants. J Pediatr Surg 36:870–876. 10.1053/jpsu.2001.23958 11381415 10.1053/jpsu.2001.23958

[7] Van Der Zwan YG, Biermann K, Wolffenbuttel KP et al (2015) Gonadal maldevelopment as risk factor for germ cell cancer: towards a clinical decision model. Eur Urol 67:692–701. 10.1016/j.eururo.2014.07.011 25240975 10.1016/j.eururo.2014.07.011

[8] Raicevic M, Saxena A (2018) Laparoscopic management of Müllerian duct remnants in the paediatric age: evidence and outcome analysis. J Minim Access Surg 14:95–98. 10.4103/jmas.JMAS_213_16 28782742 10.4103/jmas.JMAS_213_16 PMC5869986

[9] Ikoma F, Shima H, YABUMOTO H (1985) Classification of enlarged prostatic utricle in patients with Hypospadias. Br J Urol 57:334–337. 10.1111/j.1464-410X.1985.tb06356.x 4005502 10.1111/j.1464-410x.1985.tb06356.x

[10] Furuya R, Furuya S, Kato H et al (2008) New classification of midline cysts of the prostate in adults via a transrectal ultrasonography-guided opacification and dye-injection study. BJU Int 102:475–478. 10.1111/j.1464-410X.2008.07472.x 18284411 10.1111/j.1464-410X.2008.07472.x

[11] Chavhan GB, Parra DA, Oudjhane K et al (2008) Imaging of ambiguous genitalia: classification and diagnostic approach. Radiographics 28:1891–1904. 10.1148/rg.287085034 19001646 10.1148/rg.287085034

[12] Okur H, Gough DCS (2003) Management of Müllerian duct remnants. Urology 61:634–637. 10.1016/S0090-4295(02)02418-4 12639661 10.1016/s0090-4295(02)02418-4

[13] Mansour M, Fattal A, Ouerdane Y et al (2021) A 35-year-old father with persistent Mullerian duct syndrome and seminoma of the right undescended testis: a rare case report. Surg Case Rep 7:1–6. 10.1186/s40792-021-01354-w 34958435 10.1186/s40792-021-01354-w PMC8712282

[14] Cools M, Wolffenbuttel KP, Drop SLS et al (2011) Gonadal development and tumor formation at the crossroads of male and female sex determination. Sex Dev 5:167–180. 10.1159/000329477 21791949 10.1159/000329477

[15] Cools M, Drop SLS, Wolffenbuttel KP et al (2006) Germ cell tumors in the intersex gonad: old paths, new directions, moving frontiers. Endocr Rev 27:468–484. 10.1210/er.2006-0005 16735607 10.1210/er.2006-0005

[16] Thiel DD, Erhard MJ (2005) Uterine adenosarcoma in a boy with persistent Müllerian duct syndrome: first reported case. J Pediatr Surg 40:e29–e31. 10.1016/j.jpedsurg.2005.05.071 16150330 10.1016/j.jpedsurg.2005.05.071

[17] Vandersteen DR, Chaumeton AK, Ireland K, Tank ES (1997) Surgical management of persistent Mullerian duct syndrome. Urology 49:941–945. 10.1016/S0090-4295(97)00104-0 9187705 10.1016/s0090-4295(97)00104-0

[18] Shenoy K, Dama S, Makam R (2023) Persistent Müllerian duct syndrome: a surgical surprise and management during laparoscopic transabdominal pre-peritoneal repair. J Minim Access Surg 19:155–157. 10.4103/jmas.jmas_368_21 36722541 10.4103/jmas.jmas_368_21 PMC10034806

[19] Verma SK, Shetty BS, Kanth L (2006) A boy with acute urinary retention: a Mullerian duct remnant (2006: 3b). Eur Radiol 16:1401–1403. 10.1007/s00330-005-0116-y 16607498 10.1007/s00330-005-0116-y

[20] Yeung CK, Sihoe JDY, Tam YH, Lee KH (2001) Laparoscopic excision of prostatic utricles in children. BJU Int 87:505–508. 10.1046/j.1464-410X.2001.00132.x 11298044 10.1046/j.1464-410x.2001.00132.x

[21] Willetts IE, Roberts JP, MacKinnon AE et al (2003) Laparoscopic excision of a prostatic utricle in a child. Pediatr Surg Int 19:557–558. 10.1007/s00383-003-0993-6 13680292 10.1007/s00383-003-0993-6

[22] Barrena S, Aguilar R, Olivares P et al (2010) Resección laparoscópica del utrículo prostático en niños [Laparoscopic resection of the prostatic utricle in children]. Cir Pediatr 23:15–18. PMID: 20578571 20578571

[23] Ikoma F, Hohenfellner R, Yamamoto S (2014) Die kaudale Migration des Verumontanums und Erweiterung des Utrikulus bei der Hypospadie [Caudal migration of the verumontanum and enlargement of the utricle in hypospadias]. Urologe A 53:1344–1349. 10.1007/s00120-014-3597-y 25142789 10.1007/s00120-014-3597-y

[24] Ahmed SF, Achermann JC, Arlt W et al (2016) Society for endocrinology UK guidance on the initial evaluation of an infant or an adolescent with a suspected disorder of sex development (revised 2015). Clin Endocrinol (Oxf) 84:771–788. 10.1111/CEN.12857 26270788 10.1111/cen.12857 PMC4855619

[25] Marei MM, Cserni T (2023) Vas deferens-sparing laparoscopic removal of a large prostatic utricle—video display and technical report, highlighting anatomical considerations. Videourology 37(1). 10.1089/vid.2022.0030

[26] Lima M, Aquino A, Dòmini M et al (2004) Laparoscopic removal of Müllerian duct remnants in boys. J Urol 171:364–368. 10.1097/01.ju.0000102321.54818.53 14665932 10.1097/01.ju.0000102321.54818.53

[27] Lima M, Maffi M, Di Salvo N et al (2018) Robotic removal of Müllerian duct remnants in pediatric patients: our experience and a review of the literature. Pediatr Med Chir 40:27–30. 10.4081/pmc.2018.182 10.4081/pmc.2018.182 29871477

[28] Parelkar SV, Gupta RK, Oak S et al (2009) Laparoscopic management of persistent Mullerian duct syndrome. J Pediatr Surg 44:e1–e3. 10.1016/j.jpedsurg.2009.05.033 19735801 10.1016/j.jpedsurg.2009.05.033

[29] Palma I, Garibay N, Pena-Yolanda R et al (2013) Utility of OCT3/4, TSPY and β-catenin as biological markers for gonadoblastoma formation and malignant germ cell tumor development in dysgenetic gonads. Dis Markers 34:419–424. 10.3233/DMA-130972 23396295 10.3233/DMA-130972 PMC3810105

[30] Farikullah J, Ehtisham S, Nappo S et al (2012) Persistent Müllerian duct syndrome: lessons learned from managing a series of eight patients over a 10-year period and review of literature regarding malignant risk from the Müllerian remnants. BJU Int 110:E1084–E1089. 10.1111/j.1464-410X.2012.11184.x 22540537 10.1111/j.1464-410X.2012.11184.x

[31] Sancar S, Ozcakir E, Kaya M (2018) Management of the patients with persistent Müllerian duct syndrome: Is the ultimate goal testicular descent? Turk J Urol 44:166–171. PMID: 29511588، PMCID: PMC5832380 29511588 10.5152/tud.2018.33407 PMC5832380

[32] Abd El-Monsif MS, Arafa N, Marei MM et al (2024) Laparoscopy versus ultrasonography for the evaluation of Müllerian duct remnants in male patients with disorder of sex differentiation. Egypt Pediatric Assoc Gaz 72:28. 10.1186/s43054-024-00256-4

[33] Wei CH, Wang NL, Ting WH et al (2014) Excision of Mullerian duct remnant for persistent Mullerian duct syndrome provides favorable short- and mid-term outcomes. J Pediatr Urol 10:929–933. 10.1016/j.jpurol.2014.01.012 24594348 10.1016/j.jpurol.2014.01.012

[34] El-Gohary MAA (2003) Laparoscopic management of persistent Müllerian duct syndrome. Pediatr Surg Int 19:533–536. 10.1007/s00383-003-0984-7 13680290 10.1007/s00383-003-0984-7

[35] Goruppi I, Avolio L, Romano P et al (2015) Robotic-assisted surgery for excision of an enlarged prostatic utricle. Int J Surg Case Rep 10:94–96. 10.1016/j.ijscr.2015.03.024 25818371 10.1016/j.ijscr.2015.03.024 PMC4430202

[36] Raza J, Warne GL (2012) Disorders of Sexual Development. In: Elzouki AY, Harfi HA, Nazer HM, Stapleton FB, Oh W, Whitley RJ (eds) Textbook of Clinical Pediatrics. Springer, Berlin, Heidelberg. pp 3649-74. 10.1007/978-3-642-02202-9_383

[37] Brandli DW, Akbal C, Eugsster E et al (2005) Persistent Mullerian duct syndrome with bilateral abdominal testis: surgical approach and review of the literature. J Pediatr Urol 1:423–427. 10.1016/j.jpurol.2005.03.011 18947583 10.1016/j.jpurol.2005.03.011

[38] Krstić ZD, Smoljanić Ž, Vukanić D et al (2000) True hermaphroditism: 10 years’ experience. Pediatr Surg Int 16:580–583. 10.1007/s003830000415 11149399 10.1007/s003830000415

[39] Mouafo Tambo FF, Dahoun S, Kamadjou C et al (2016) Mixed gonadal dysgenesis in Yaoundé: a preliminary experience about three cases. Afr J Paediatr Surg 13:145–149. 10.4103/0189-6725.187822 27502884 10.4103/0189-6725.187822 PMC11639613

