# Supplementary material for: Excision versus division of Müllerian duct remnants in male disorders of sexual development and differentiation: a prospective study to generate anatomical assessment criteria
Source: Pediatr Surg Int. 2025 Jul 30;41(1):238. doi: 10.1007/s00383-025-06079-7 (PMC12310905; doi:10.1007/s00383-025-06079-7)
Supplement: Supplementary file 1 — (DOCX 237 KB): Supplemental Figure (SF1): Laparoscopic Setup: Position of the patient and the surgical team. S: Surgeon, SA/C: Surgical assistant/Camera holder, SN:Scrub nurse, AN: Anaesthesiologist [file 383_2025_6079_MOESM1_ESM.docx]

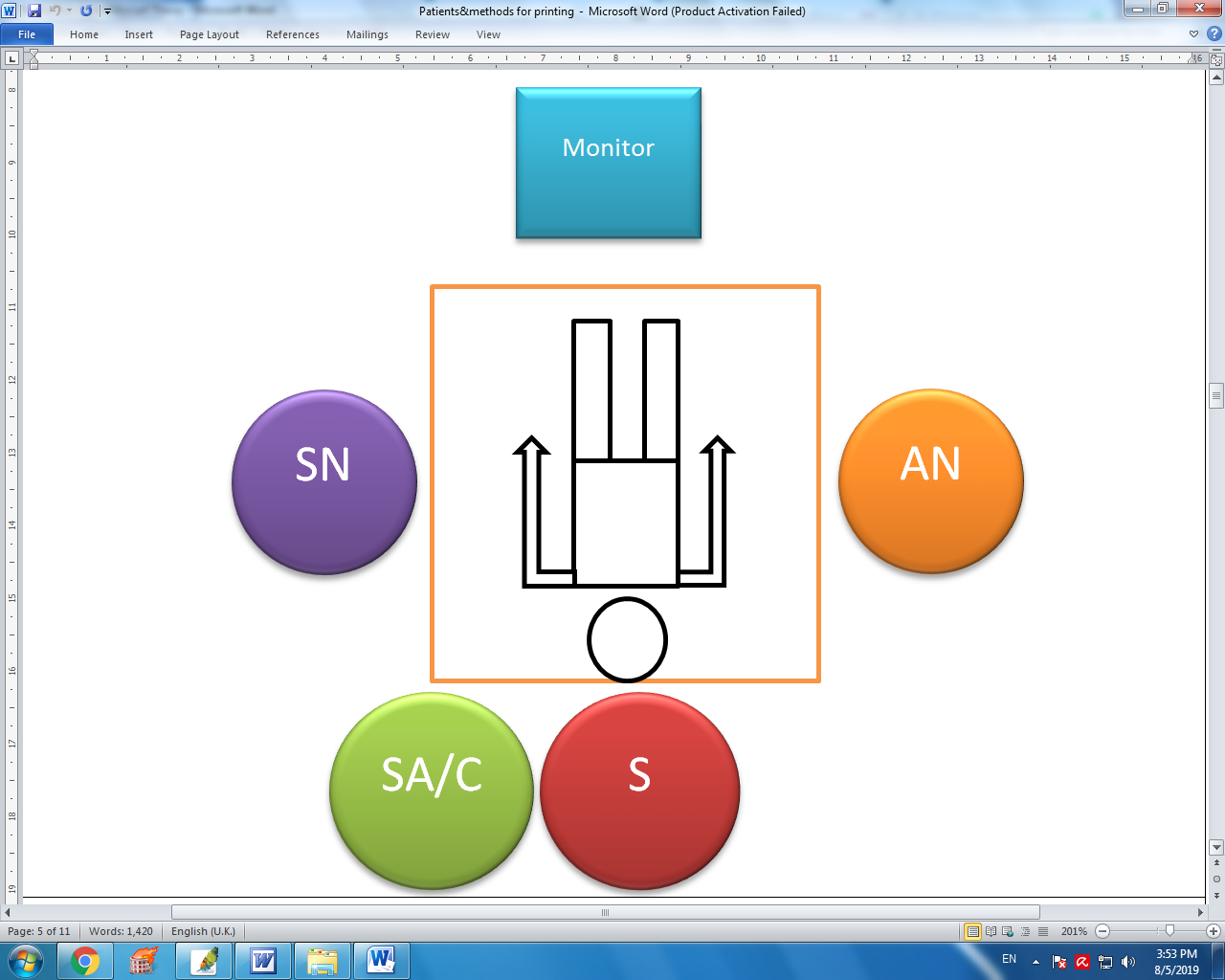
**Supplemental Figure (SF1): Laparoscopic Setup: Position of the patient and the surgical team.**

*S*–Surgeon; *SA/C*–Surgical Assistant/Camera Holder; *SN*–Scrub Nurse; *AN*–Anaesthetist (*SN* and *AN* may be reversed, depending on theatre setup).
