# Supplementary material for: Excision versus division of Müllerian duct remnants in male disorders of sexual development and differentiation: a prospective study to generate anatomical assessment criteria
Source: Pediatr Surg Int. 2025 Jul 30;41(1):238. doi: 10.1007/s00383-025-06079-7 (PMC12310905; doi:10.1007/s00383-025-06079-7)
Supplement: Supplementary file 2 — (DOCX 21 KB): Supplemental Table (ST1): Examination Data in All Initially Enrolled Cases.Examination Data. Data presented as number (percentage). Percentages are calculated inreference to all initially enrolled cases [file 383_2025_6079_MOESM2_ESM.docx]

**Supplemental Table (ST1): Examination Data in All Initially Enrolled Cases. Data presented as number (percentage)^¤^.**

| **External genital examination** | | **Total (N = 20)** |
| --- | --- | --- |
| Length of phallus (cm) | Mean ± SD | 3.63 ± 0.94 |
|  | Range | 2.5–6 |
| Micropenis: Less than 2.5 SD below the mean for age | Absent | 15 (75.0%) |
|  | Present | 5 (25.0%) |
| Hypospadias | Absent | 3 (15.0%) |
|  | Present | 17 (85.0%) |
|  | Mid-penile | 1 (5.9%) |
|  | Proximal penile | 1 (5.9%) |
|  | Penoscrotal | 7 (41.2%) |
|  | Scrotal | 7 (41.2%) |
|  | Perineal | 1 (5.9%) |
| Labioscrotal folds | Fused | 12 (60.0%) |
|  | Bifid scrotum | 8 (40.0%) |
| Undescended gonads | Unilateral | 8 (40.0%) |
|  | Bilateral | 12 (60.0%) |
| Rt: Right gonad  Lt: Left gonad | Palpable at the normal scrotal position | Rt: 4 (20.0%)  Lt: 4 (20.0%) |
|  | Palpable at the neck of the scrotum | Rt: 1 (5.0%)  Lt: 0 (0.0%) |
|  | Palpable at the Inguinal region | Rt: 2 (10.0%)  Lt: 2 (10.0%) |
|  | Impalpable | Rt: 13 (65.0%)  Lt: 14 (70.0%) |

^¤^Percentages are calculated in reference to all initially enrolled cases.
