# Supplementary material for: Excision versus division of Müllerian duct remnants in male disorders of sexual development and differentiation: a prospective study to generate anatomical assessment criteria
Source: Pediatr Surg Int. 2025 Jul 30;41(1):238. doi: 10.1007/s00383-025-06079-7 (PMC12310905; doi:10.1007/s00383-025-06079-7)
Supplement: Supplementary file 3 — (DOCX 21 KB): Supplemental Table (ST2): Chromosomal and Endocrinal Status of Surgically Operated Cases (N =18). Data presented as number (percentage). †Sig.: p-value > 0.05: Non-significant (NS); p-value < 0.05: Significant (S); p-value < 0.01: Highly significant (HS); *Chi-square test. Percentages are calculated in reference to the subgroup, i.e. Excision = 12 patients and Division = 6 patients [file 383_2025_6079_MOESM3_ESM.docx]

**Supplemental Table (ST2): Chromosomal and Endocrinal Status of Surgically Operated Cases (N=18). Data presented as number (percentage)^¤^.**

| **Investigation** | **Findings** | **Excision**  **(*n*=12)** | **Division**  **(*n*=6)** | **Test value^*^** | ***P* value** | **Sig.^†^** |
| --- | --- | --- | --- | --- | --- | --- |
| Karyotyping | 46, XY | 9 (75.0%) | 5 (83.3%) | 4.154^*^ | 0.245 | NS |
|  | 46, XX | 0 (0%) | 1 (16.7%) |  |  |  |
|  | 46, XY \ 46, XX | 0 (0%) | 0 (0%) |  |  |  |
|  | 46, XY \ 46, XO | 3 (25.0%) | 0 (0%) |  |  |  |
| AMH level | Average for age | 10 (83.3%) | 2 (33.3%) | 4.500^*^ | 0.034 | S |
|  | Low for age | 2 (16.7%) | 4 (66.7%) |  |  |  |

^†^Sig.: *p*-value > 0.05: Non-significant (NS); *p*-value < 0.05: Significant (S); *p*-value < 0.01: Highly significant (HS); ^*^Chi-square test; ^¤^Percentages are calculated in reference to the subgroup, i.e. Excision = 12 patients and Division = 6 patients; *AMH*–Anti-Müllerian hormone.
