# Supplementary material for: Excision versus division of Müllerian duct remnants in male disorders of sexual development and differentiation: a prospective study to generate anatomical assessment criteria
Source: Pediatr Surg Int. 2025 Jul 30;41(1):238. doi: 10.1007/s00383-025-06079-7 (PMC12310905; doi:10.1007/s00383-025-06079-7)
Supplement: Supplementary file 4 — (DOCX 21 KB): Supplemental Table (ST3): Gonadal Status and Positions by Laparoscopy. Data presented as number (percentage). Percentages are calculated in reference to all initially enrolled cases [file 383_2025_6079_MOESM4_ESM.docx]

**Supplemental Table (ST3): Gonadal Status and Positions by Laparoscopy. Data presented as number (percentage)^¤^.**

| **Gonadal status and positions by laparoscopy** | **Right gonad** | **Left gonad** | **Total** |
| --- | --- | --- | --- |
| Vas deferens and vessels entering the inguinal canal | 9 (45.0%) | 4 (20.0%) | 13 (32.5%) |
| Fallopian tube entering the inguinal canal | 1 (5.0%) | 1 (5.0%) | 2 (5.0%) |
| Gonad at internal ring (peeping) | 1 (5.0%) | 1 (5.0%) | 2 (5.0%) |
| Medial to iliac vessels | 5 (25.0%) | 4 (20.0%) | 9 (22.5%) |
| Lateral to iliac vessels | 2 (10.0%) | 5 (25.0%) | 7 (17.5%) |
| Related/Attached to the ipsilateral kidney | 0 (0.0%) | 1 (5.0%) | 1 (2.5%) |
| Related/Attached to the MDR | 1 (5.0%) | 2 (10.0%) | 3 (7.5%) |
| Intra-abdominal gonads | 9 (45.0%) | 13 (65.0%) | 22 (55.0%) |
| Present gonads (at enrolment/inclusion) | 19 (95.0%) | 18 (90.0%) | 37 (92.5%) |
| Absent gonads (at enrolment/inclusion) | 1 (5.0%) | 2 (10.0%) | 3 (7.5%) |

^¤^Percentages are calculated in reference to all initially enrolled cases; *MDR*–Müllerian duct remnant.
