# Supplementary material for: Excision versus division of Müllerian duct remnants in male disorders of sexual development and differentiation: a prospective study to generate anatomical assessment criteria
Source: Pediatr Surg Int. 2025 Jul 30;41(1):238. doi: 10.1007/s00383-025-06079-7 (PMC12310905; doi:10.1007/s00383-025-06079-7)
Supplement: Supplementary file 5 — (DOCX 33 KB): Supplemental Table (ST4): Karyotype-Specific Surgical Decision Factors [file 383_2025_6079_MOESM5_ESM.docx]

**Supplemental Table (ST4): Karyotype-Specific Surgical Decision Factors.**

| **Karyotype** | **No. of Cases** | **Surgical Approach** | **Vas Deferens Relation** | **Rationale** |
| --- | --- | --- | --- | --- |
| 46, XY Ovotesticular DSD | 4 | Excision | 2 absent,  2 unilateral with clear course. | No or clear unilateral relation allowed safe excision. |
| 46, XY MGD | 3 | Excision | 1 absent,  2 unilateral with clear course. | Favourable anatomy enabled excision. |
| 46, XY / 45, XO MGD | 3 | Excision | 1 absent,  2 unilateral with clear course. | Safe dissection possible due to clear course. |
| 46, XY under-virilised (non-CAH) | 4 | 2 Excision, 2 Division | 2 absent (excision),  2 bilateral unclear (division). | Approach dictated by anatomical clarity. |
| 46, XY PMDS | 3 | Division | 2 bilateral and complex,  1 high vas deferens entry. | Division chosen to preserve vas integrity. |
| 46, XX Male | 1 | Division | Vas deferens coursing along the MDR wall. | Close relation precluded safe excision. |

*MGD*–Mixed gonadal dysgenesis; *CAH*–Congenital adrenal hyperplasia; *PMDS*–Persistent Müllerian duct syndrome.
